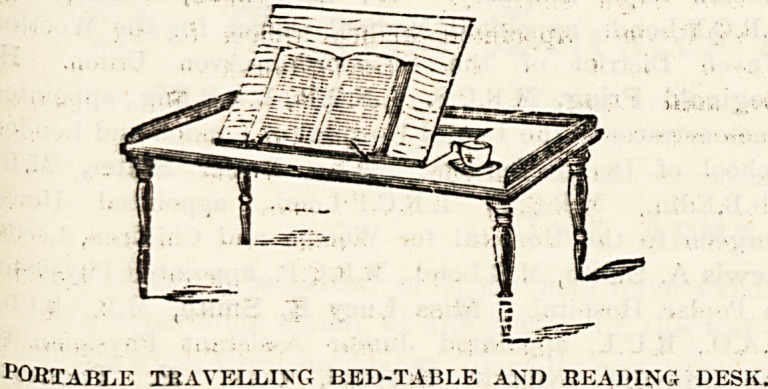# Practical Departments

**Published:** 1901-04-20

**Authors:** 


### PRACTICAL DEPARTMENTS.

### INVALID FURNITURE.

The energies of Messrs. Farmer Lane and Co. have during the past year been devoted to the perfecting of their specialities in invalid furniture, and the greatest care has been taken to secure the maximum of ease, comfort, and durability, combined with appearance. The walking-machine, or "go-car," has recently had a seat added to it, and with its rubber-tyred wheels and crutches adjustable to any height, should be a valuable aid to those patients in hospitals, or wounded soldiers from South Africa, who have partially or entirely lost the use of their lower limbs. The self-propelling chairs made by this firm are almost too well known to need description; they are in use in some of the largest of our hospitals. A very useful and ingenious variety is one which has a couple of sliding boards hidden under the seat; these can be pulled out to support the patient's legs. A sliding foot-rest is hidden in the same manner. These chairs are very easily propelled by the patient, and are made in great variety.

The combination couch, lounge, and easy chair is simple in construction, and thoroughly well made and finished, and Messrs. Farmer, Lane and Co., who are the inventors, claim for it that in each form it is as perfect in its action as if made in that only. It is easily portable, and especially adapted for travelling, since it folds into a compact form. It has a unique arrangement by means of which the seat can be slightly inclined backwards; this is a great advantage when the patient is old or very feeble, as it prevents him from slipping out of the chair while asleep. The luxurious "adjustable nurse chair" must receive a word of notice; the stuffing is all hair, and it is well provided with springs; the upholstering is in rep, velvet, &c., as required; the leg rest draws out from under the seat, and can be fixed at any inclination, while the person occupying the chair can alter the angle of the back without getting up. By means of specially constructed large brass wheel castors it can be moved from one room to another. Some very convenient reading stands are also to be seen at 79 New Oxford Street; they can be supplied either with or without a small circular table as well as the book-rest, and one really very luxurious stand has not only a table but two writing or reading rests. These "reading machines" are very suitable for private patients. A very useful present, and an inexpensive one as well, is a polished pine bed-table, with a movable desk for reading or writing; and indeed the enormous variety of invalid furniture both for hospital and home use made by this firm would seem to make illness almost a luxury. The long list of hospitals recently supplied proves that the goods manufactured in

PORTABLE TRAVELLING BED-TABLE AND READING DESK.

Messrs. Farmer Lane's workshops have a solid and wide reputation. Invalid requisites, such as bath chairs, water-beds, &c., are lent to non-infectious cases at reasonable rates. The arrangements for free delivery to any station in the United Kingdom enables country customers to buy satisfactory articles at the same cost as if living in London, while at the same time ensuring the highest quality of workmanship.

## Figures and Tables

**Figure f1:**
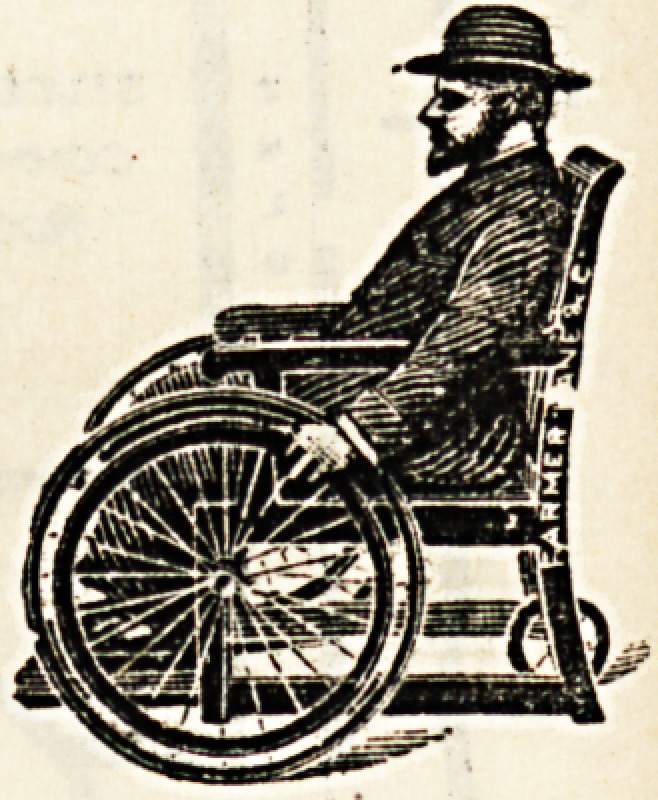


**Figure f2:**